# Diagnosis of Bardet-Biedl Syndrome in Consecutive Pregnancies Affected with Echogenic Kidneys and Polydactyly in a Consanguineous Couple

**DOI:** 10.1155/2013/159143

**Published:** 2013-03-04

**Authors:** Tieneka M. Baker, Erica L. Sturm, Clesson E. Turner, Scott M. Petersen

**Affiliations:** ^1^Department of Obstetrics and Gynecology, Walter Reed National Military Medical Center, 8901 Wisconsin Avenue, Bethesda, MD 20889, USA; ^2^Prenatal Assessment Center, Walter Reed National Military Medical Center, Bethesda, MD 20889, USA; ^3^Medical Genetics, Walter Reed National Military Medical Center, Bethesda, MD 20889, USA; ^4^Maternal-Fetal Medicine, Walter Reed National Military Medical Center, Bethesda, MD 20889, USA

## Abstract

Bardet-Biedl syndrome (BBS) is an autosomal recessive ciliopathic human genetic disorder with variable expression that is difficult to diagnose in pregnancy without known risk factors. Homozygosity testing has been shown to be a useful tool in identifying BBS mutations and candidate genes in affected individuals. We present the first case of prenatal diagnosis of BBS in consecutive pregnancies aided by homozygosity testing via SNP microarray analysis. This case demonstrates a novel approach to the evaluation of recurrent echogenic kidneys in consanguineous couple with no significant family history.

## 1. Introduction

Postnatal diagnosis of Bardet-Biedl syndrome (BBS; OMIM number 209900) is generally established by clinical findings. Primary features of the disease include postaxial polydactyly, renal anomalies, rod-cone dystrophy, truncal obesity, learning disabilities, and hypogonadism in males or genital abnormalities in females. Secondary features include speech delay, developmental delay, behavioral abnormalities, eye abnormalities, ataxia/poor coordination, mild hypertonia, diabetes mellitus, orodental abnormalities, cardiovascular anomalies, hepatic involvement, craniofacial dysmorphism, Hirschsprung disease, and anosmia [1]. BBS demonstrates highly variable expression, even among affected siblings, making the diagnosis difficult and often delayed. 

There are several reports outlining the use of ultrasound in the diagnosis of BBS in the antenatal period. They are primarily retrospective reviews or involve an at-risk fetus with an affected sibling. However, echogenic kidneys observed during level II ultrasound should raise suspicion of possible BBS. The differential diagnosis of fetal echogenic kidneys is broad and is reliant on whether or not associated anomalies are visualized [2]. Within the associated genetic syndromes, there is considerable overlap amongst the presenting features. A thorough family history should be obtained and may be useful. However, many of the associated syndromes are autosomal recessive and relatively uncommon; therefore, recurrence may be distant if present. 

There are currently 15 genes that are known to be associated with BBS [3]. Mutations are primarily identified through sequence analysis, targeted mutation analysis, or deletion/duplication analysis once the clinical diagnosis is made. Homozygosity testing via single nucleotide polymorphism (SNP) microarray analysis in individuals affected with BBS has been well established in identifying new genes and mutations in a research setting [4, 5]. To date, there are no documented cases of homozygosity testing utilizing SNP analysis in assisting with prenatal diagnosis of BBS after miscarriage or abortion in a family without a documented family history of the disease.

## 2. Case

A 28-year-old patient was referred at 12-week gestation of her first pregnancy for genetic counseling regarding consanguinity. The patient and her 43-year-old husband are first cousins, once removed. Her husband has three healthy children from a previous relationship. The family history was otherwise unremarkable and noncontributory. The significance of common ancestry and the risk of autosomal recessive conditions were discussed and level II ultrasound was recommended. 

At 20-week gestation, she returned for an anatomic survey and the fetus was found to have multiple renal cysts, suspicious for autosomal recessive polycystic kidney disease (ARPKD). The patient declined amniocentesis and elected termination of pregnancy based on the presumed poor prognosis. Dilation and extraction (D&E) was performed at 20 weeks at an outside facility where termination was able to be legally performed and the obtained tissue was transported to the National Naval Medical Center (NNMC) for partial autopsy of products of conception. Tissue biopsies were sent for fibroblast culture. Chromosome analysis demonstrated a 46,XY karyotype, and sequence analysis of *PKHD1* for ARPKD was negative. Limited inspection of fetal remains confirmed bilateral renal cysts and identified bilateral postaxial hexadactyly in the lower extremities, suspicious for Meckel-Gruber syndrome although no evidence of occipital defect was noted. Sequence analysis of *MKS1, TMEM67, CC2DA*, *RFGRIP1L*, and *CEP290* for Meckel-Gruber syndrome was performed and failed to detect a causative mutation. Evaluation for other ciliopathies was considered, but no further testing was performed without additional clues to narrow the differential and the cost associated with additional single gene analysis in this setting. In the absence of a specific diagnosis, the patient was counseled regarding an expected 25% recurrence risk given the findings, their known consanguinity, and autosomal recessive inheritance of most ciliopathies. 

The patient presented with a second pregnancy soon thereafter. Nuchal translucency evaluation was performed at 12-week gestation and was within normal limits to include a limited anatomic survey. Early attempt at an anatomy assessment was performed at 16 weeks and again identified bilateral multicystic kidneys and polydactyly of the lower extremities, clearly visible on transvaginal ultrasound. The subsequent fetus was female. The patient again elected pregnancy termination by D&E at an outside facility. Fetal remains were again transported for fibroblast tissue culture. After multidisciplinary discussion of the case, pursuit of homozygosity testing was recommended to narrow the range of regions of suspicion. 

Homozygosity mapping has been demonstrated as a useful tool in both research and clinical genetics [6]. It involves analysis of single nucleotide polymorphisms (SNPs) in an attempt to identify two copies of the same mutation segregating on the same haplotype. This is particularly attractive for families of known consanguinity in which a common ancestral mutation is likely to be shared by both parents. Homozygosity testing of our family's samples was performed at Children's Hospital of Philadelphia using the Illumina HumanQuad610 BeadChip, which contains greater than 500,000 autosome loci, 15,000 loci from the X and 2,000 loci from the Y chromosome. The male fetus was identified to have 7 regions of homozygosity. The female fetus was identified to have 9 regions of homozygosity. Four regions of homozygosity were common to both fetuses (see Table 1), including a 5.75 Mb sequence on chromosome 14 (chr14:85,720,831-91,471,338), which is the location of *TTC8*, one of the genes responsible for BBS (BBS8). Further sequence analysis of *TTC8* performed at PreventionGenetics identified homozygous deletion c.624+1_624+2delGT splicing mutation, which had been previously reported as a single heterozygous mutation in a BBS patient and is expected to be pathogenic [7]. After disclosure of the results, the family indicated that they intend to use the mutation identified to allow for preimplantation genetic diagnosis (PGD) of BBS in an attempt to prevent this condition from recurring in future pregnancies.

## 3. Comment

BBS is a rare condition that has an extremely variable presentation, and its features can be quite debilitating. With earlier exposure to prenatal care along with the increased accessibility and use of ultrasound in the antenatal period, more anomalies are being identified earlier in pregnancy. In patients that are at high risk for certain disorders, these anomalies can provide sufficient evidence that a particular disease is present without the need for further testing. Dar et al. describe the diagnosis of BBS via a targeted ultrasound at 16 weeks revealing postaxial polydactyly in a pregnancy of a woman with two prior children affected with the disorder [8]. With the presumed diagnosis the patient decided to terminate the pregnancy. Cassart et al. looked retrospectively at 127 patients with hyperechoic kidneys, targeting 11 who ultimately were diagnosed with BBS via clinical manifestations [9]. They confirmed that in a family with known history of BBS, ultrasound findings of polycystic kidneys with or without polydactyly confer a very high likelihood of recurrence. Through their review they noted that BBS should be considered as a differential diagnosis in those with echogenic kidneys without a known family history. This information should be included in further evaluations as well as genetic counseling to help guide decision about invasive testing and what method of genetic analysis should be pursued.

Particularly in patients without a known family history or prior diagnosis, ultrasound findings without definitive diagnosis or prognosis, further genetic testing may be useful. Karmous-Benailly et al. evaluated 13 patients with polycystic kidneys and polydactyly by prenatal ultrasound for evidence of mutations related to BBS [10]. Six patients were found to have homozygous mutations, three with compound heterozygous mutations, and four without any mutations identified. The shortcoming of this study was that only known BBS mutations were tested for, and it is expected that at least 20% of patients with BBS will not have one of these mutations. 

Homozygosity testing has been used to identify new mutations in known genes and to identify new candidate genes in affected individuals with a clinical diagnosis of BBS. Pereiro et al. describe its use for the identification of the affected genes in nine families from Spain [5]. Six disease-causing mutations were identified with five notable for novel sequence variants. The remaining three revealed homozygous candidate regions which could lead to the identification of new mutations related to the BBS diagnosis. The benefits of homozygosity testing, especially utilizing SNP analysis, in rare autosomal recessive disorders such as BBS allow for a faster and more cost-effective means of searching the genome for homozygous loci, essentially narrowing down possible causative genes.

Although the studies mentioned have similar aspects, our case demonstrates a novel means to obtaining the diagnosis of BBS from any other previously described. Ultrasound is a known means of identifying patients at high risk for BBS with findings of polycystic kidneys as well as polydactyly. Homozygosity testing has been shown to be a useful tool in identifying BBS mutations in affected individuals. This case demonstrates the use of these two tools to aid in the diagnosis of BBS after consecutive terminations of pregnancy with limited clinical information. Homozygosity testing via SNP microarray analysis allowed the identification of common regions of homozygosity, which made further molecular studies more accessible. Molecular diagnosis now allows the couple the option of earlier prenatal diagnosis with chorionic villous sampling or use of PGD. A diagnosis of BBS also carries a different prognosis than other related ciliopathies expected to be lethal, such as Meckel-Gruber syndrome. Specific diagnosis is not only helpful in optimizing the couple's reproductive choices but also is also relevant in the counseling of similarly affected pregnancies in outlining appropriate expectations.

The management of our case was further enhanced by the information gained from limited autopsy review despite the method of pregnancy termination and having samples from two separate affected pregnancies. While it is clear that autopsy following termination by early induction would be preferred, if accessible and feasible, anatomic inspection may still be useful following D&E. Also, with the increasing utility of SNP testing, we may have finally reached a point where DNA banking from fetuses with anomalies may warrant further consideration and may soon be useful.

## Figures and Tables

**Table 1:** 

Regions of homozygosity in affected siblings	
6.32 Mb on chromosome 11 (chr11: 48, 920, 995-55, 240, 228)	
3.19 Mb on chromosome 11 (chr11: 74, 836, 081-78, 022, 487)	
12.18 Mb on chromosome 12 (chr12: 115, 621, 170-127, 797, 029)	
5.75 Mb on chromosome 14 (chr14: 85, 720, 831-91, 471, 338)	
